# Building capacity in primary care rehabilitation clinical practice guidelines: a South African initiative

**DOI:** 10.1186/s12961-018-0368-z

**Published:** 2018-09-29

**Authors:** Q. Louw, K. Grimmer, J. M. Dizon, S. Machingaidze, H. Parker, D. Ernstzen

**Affiliations:** 1 Department of Physiotherapy, Faculty of Medicine and Health Sciences, Stellenbosch University, Francie van Zijl Drive, Tygerberg, Cape Town, 7505 South Africa; 2 Clinical Teaching and Education Centre, College of Nursing and Health Sciences, Flinders University, Daw Park, South Australia 5041; 3 International Centre for Allied Health Evidence, University of South Australia, City East Campus, Adelaide, 5000 Australia; 4 South African Cochrane Centre, South African Medical Research Council, Francie van Zijl Drive, Parow Valley, Cape Town, 7505 South Africa

## Abstract

**Background:**

The large number of South Africans with disability who cannot access good quality rehabilitation presents a public health and human rights challenge. A cost-effective, efficient approach is required to address this. Implementation of high-quality, contextually relevant clinical practice guidelines (CPGs) could be a solution; however, this requires significant investment in innovative capacity-building.

**Methods:**

A qualitative descriptive national study was conducted to explore the perspectives of South African stakeholders in rehabilitation, regarding CPG capacity-building. Twenty rehabilitation professionals (physiotherapists, occupational therapists, speech language therapists, podiatrists, rehabilitation managers or directors) were interviewed. Transcribed interview data were analysed using a deductive content analysis approach, mapping findings to an international capacity-building framework to produce new knowledge.

**Results:**

Capacity-building is required in content, purpose and construction of locally relevant CPGs, as well as personal, workforce and systems capacity. Principles and strategies were derived to underpin implementation of CPGs that are user friendly, context specific, relevant to the needs of end-users, and achievable within available resources. Collaboration, networks and communication are required at national, provincial and regional level, within and between sectors. A central agency for CPG methods, writing, implementation and evaluation is indicated.

**Conclusion:**

South African rehabilitation can benefit from a multi-level CPG capacity-building focusing on performance, personal, workforce and systems issues.

## Background

Rehabilitation based on best-available evidence has the potential to cost-effectively optimise health outcomes, quality of life and well-being [1]. It is estimated that 15% of the world’s population currently has some form of disability that requires rehabilitation and/or assistive technology [2]. Access to effective rehabilitation is a basic human right [3]. Despite the huge need in South Africa, there are millions without any access to rehabilitation [4]. Moreover, those with access often receive inadequate, ineffective or non-evidence-based care, which wastes scarce rehabilitation resources [1–3]. With no anticipated lifting of current economic constraints in South Africa, it is unlikely that rehabilitation services will be increased [4]. Thus, new thinking is required to ensure that available resources are distributed equitably and to optimise benefits.

WHO recognises the need to reform healthcare, and is advocating for rehabilitation to be included into universal health coverage [2, 3]. South Africa is currently embarking on implementing the country’s first national health insurance scheme [5]. The challenge for South African rehabilitation professionals is to ensure that the national insurance scheme incorporates rehabilitation, and that it is accessible by all who need it. One barrier to addressing this challenge is the absence of national evidence-based, rehabilitation clinical practice guidelines (CPGs) [4].

Effective production and implementation of CPGs relates to local capacity [2, 3]. Capacity may variably reflect workforce, training and knowledge, funding, policy and communication. Capacity and capacity improvement are complex notions that can mean different things to different people. Given the urgent challenges for those involved in rehabilitation in South Africa to meet the requirements of the South African national health insurance scheme, it is timely to consider current and future South African rehabilitation capacity. Rehabilitation in South Africa is largely provided by allied health (AH) disciplines of physiotherapy, occupational therapy, and speech and language therapy, across all levels of healthcare [4]. To assist these health professionals to provide the highest quality care possible within local contexts, it is important to identify the barriers they face, and the facilitators they can draw on, when implementing best evidence-based rehabilitation.

Discussions on understanding and improving capacity began in the 1970s, with increasing advocacy for empowerment of communities in developing countries to build capacity in public health and education [6]. To date, there is no agreed definition of the activities related to improving capacity. The terms capacity-building, or capacity development, are commonly used, yet many argue that these terms are too broad to have practical meaning within specific contexts [6]. Moreover, central to the notion of building, developing or improving capacity is flexibility and independence in achieving sustainable changes [7, 8]. James uses the terms “*capacity challenge*” and “*dimensions of issues and needs*” whilst describing the goal of capacity-building as “*to tackle problems that are related to policy and methods of development, whilst considering the potential limits and needs of the people of the country concerned*” ([7], p. xi). Building, developing or improving capacity is a complex, multidimensional activity that must be relevant to local contexts and needs. Potter and Brough [8] argue that there is a hierarchy of capacity-building needs, which must be considered for effective and sustainable outcomes. They proposed a capacity framework to assist in identifying the elements required to improve capacity in specific situations, and to understand the inter-relationships between these elements relevant to local contexts. This framework involves four levels, as follows: (1) structures, systems and roles, (2) staff and facilities, (3) skills, and (4) tools required for capacity-building. This framework offers a facilitated approach in which local knowledge becomes an essential element of effective interventions that produce sustainable capacity improvements [6–8].

To build capacity for routine evidence implementation into rehabilitation will require an understanding of the specific obstacles that inhibit change in each of these areas [6, 7]. Irrespective of which issues are involved in capacity improvement activities, behaviour change is generally required [7, 8]. There is a growing recognition of the efficiencies of using current, good quality CPGs as a ‘one-stop shop’ to inform best practices [9], as well as a growing body of knowledge about how to change behaviours to effectively implement CPGs in policy, funding and care decisions [8]. To our knowledge, there is no recommended approach for building capacity around CPG uptake in rehabilitation with the aims of enhancing equity and quality of rehabilitation care, and which could inform national insurance requirements.

As part of the South African Medical Research Council Flagship-funded South African Guidelines Excellence (SAGE) Project [10] (2015–2017), we explored a range of issues related to the use of CPGs in South African primary healthcare, by interviewing key informants in medicine, nursing and AH [11, 12]. Complex barriers to effectively writing and implementing CPGs were identified, including inadequate knowledge and resources (funding, staff shortages, staff turnover), poor professional and policy representation at provincial and national health departments, and lack of incentives, training and recognition [11, 13]. Many of the AH key informants provided insights specifically into rehabiliation, particularly identifying the critical importance of improving capacity in CPG writing and implementation as a way of improving rehabilitation quality, and facilitating inclusion of best-practice rehabilitation into the South African national health insurance scheme.

## Methods

### Aims and reporting standard

This paper reports on a subset of interview data captured in a large qualitative descriptive study of CPG use in South African primary care environments. The aim of writing this paper was to explore the issues raised regarding improving capacity to apply CPGs in rehabilitation. The reporting is framed by the COREQ statement [14].

### Ethics

Ethical approval was provided by the three collaborating institutions involved in Project SAGE: South Africa Medical Research Council (EC002–2/2014), Stellenbosch University (South Africa) (N14/02/ 008) and University of South Australia, Australia (0000034923).

### Research team

The six-person research team comprised four individuals with experience in CPG writing and implementation, who also had backgrounds in rehabilitation (KG, QL, JMD, DE), a public health epidemiologist (SM) with expertise in systems analysis, and a social scientist (HP) with expertise in qualitative methods and analysis. All team members were experienced in conducting and reporting qualitative research.

### Establishing the reference sample

At the time the research was conducted, there was no published information about primary care AH or rehabilitation CPG activity in South Africa, and no formal networks to support robust sampling [4, 11, 15]. To this end, a preliminary scoping study was conducted to establish a robust sampling framework of AH stakeholders [12]. This addressed items 10 and 11 of the COREQ criteria, namely, defining the sample and explaining how subjects were identified [14]. To define the sampling framework, we (1) searched the South African government websites for structures of departments and portfolios that included AH voices; (2) searched these same websites for CPGs that involved AH, particularly in rehabilitation; (3) searched professional AH association websites for evidence of CPG activity; (4) critiqued CPGs for priority South African primary care conditions, for evidence of AH involvement; and (5) identified stakeholder groups (which we called clusters) that had an interest in, or views on, CPG use in rehabilitation (these comprised National and provincial policy-makers, public and private sector clinicians in metropolitan, rural and remote areas, academics, facility managers in metropolitan, rural and remote areas, leaders in professional associations, members of rehabilitation forums and special interest groups, and consultants).

### Selecting the sample

We used a cluster sampling framework that supported effective, efficient and comprehensive maximum variation sampling [12, 16, 17]. We used a maximum variation sampling approach to capture the heterogeneity and breadth of knowledge available in the reference population [16]. This approach was resource efficient in ensuring that we heard different AH voices with diverse experiences, roles and knowledge of primary healthcare rehabilitation CPG activities across different geographical and work settings [12]. We collated our sample using a purposive, consecutive snowballing approach within each cluster [16, 17]. To do this, we commenced with one or two key individuals in each cluster, identified directly by SAGE team members, or from information provided on websites, or from telephoning AH professional associations, rehabilitation and disability organisations. We decided on a project-specific ‘stopping rule’ of three requests to the same person without response, before we ceased that recruitment attempt. If the initially identified individuals agreed to participate, during the interview, they were asked for names of others in that cluster (or any other relevant stakeholder group), who could assist us. If key individuals refused the initial interview request, we asked them for names of others whom we could approach. We continued this sampling approach until no further names were proposed in any stakeholder cluster.

### Data collection

We invited nominated individuals to participate in individual semi-structured interviews. The interview schedule allowed exploration of a broad range of issues related to CPG need, writing and implementation in primary healthcare settings in South Africa. Interview questions sought to obtain information on (1) existing frameworks, supports and activities that are used to develop and implement CPGs in rehabilitation, (2) current roles, skills and availability of resources available to support implementation of CPGs and what additional resources are required, (3) barriers and facilitators for the development and implementation of CPGs, and (4) the contexts in which rehabilitation CPGs are formulated and implemented. The interviewer encouraged exploration of responses using a combination of conventional interview techniques (e.g. probing questions, seeking clarification, confirming answers if required and presenting reflections) [16]. Interviews were conducted from May to September 2015. Each interview lasted for approximately an hour.

Participants provided formal written consent for audio recordings. Where participants were not comfortable providing formal consent for recording the entire interview, notes were taken during periods where information was provided ‘off the record’. This assisted the researchers to validate and contextualise the formally consented material without breaching confidence.

Researchers conducted interviews in pairs, which provided consistency and coherence in data collection, handling and analysis [16, 17]. Regarding clarity for further confirmation of responses, the encounter would consist of one lead and one supporting interviewer. Researchers remained ‘reflexive’ during interviews [16], and attempted to reproduce responses of participants for transparency and clarity or to seek further explanation. The interviewer pairs discussed each interview afterwards and identified key issues arising from it. Interview notes were kept, noting circumstances, key issues and nuances.

Once we suspected that we had reached saturation in any cluster (where nothing new was heard since the last interview), one further interview was conducted with the next participant. If no new information was forthcoming, interviewing in that cluster (not discipline) stopped at that point. However, if new information was found in that interview, further sampling and interviewing occurred until data saturation was achieved.

### Researchers’ relationship with participants

One researcher (QL) was known to eight participants, and a second researcher (KG) was also known to four of these. When inviting participants to join the research, the researchers declared their position (and prior knowledge), and the intent of the research. They clarified ways in which participant anonymity would be protected. Prior to the interview, all interviewees reviewed the information sheet, signed the consent form, and clarified issues and concerns.

### Framework for analysis

We applied an explorative, descriptive methodology to data analysis [16, 17], underpinned by a deductive reasoning approach [18]. This approach was selected after interviews were completed, and we chose the Potter and Brough four-tier hierarchy of capacity-building needs as the framework for analysis (reflecting (1) structures, systems and roles, (2) staff and facilities, (3) skills, and (4) tools). This interactive framework was developed from extensive research in the United States of America and India, and defines key capacity elements that have been applied by others in capacity development interventions [19]. This framework offered us a practical approach to considering capacity-building in the South African rehabilitation environment, where there has been minimal concerted intervention to date [4, 13, 20]. By mapping our interview findings to this framework, we were positioned to identify specific issues and propose strategies that could improve capacity in writing and implementing South African rehabilitation CPGs [7].

### Data management

All interviews were audiotaped and then independently transcribed. A manual coding process was applied first, where transcripts were examined as meaningful parts of the collective research study. The interview notes were used for validation and as *aides-de-memoir*. To establish reliability in theme identification, four researchers independently hand-analysed two randomly selected interviews (N1, N16) and discussed their findings, particularly the differences in interpretation. They then independently analysed one further interview (N18) to test agreement. During this process, the researchers started defining the key themes according to Potter’s framework [7].

For more in-depth and structured analysis, all transcripts were then transferred into a data bundle in *Atlas.ti*, a qualitative data analysis software programme [21]. The interviews were then divided between three researchers (QL, JD, HP) using a random generator calculator (MSExcel Version7), and analysed independently in *Atlas.ti* for family codes, themes and subthemes. The individual data bundles were then combined in *Atlas.ti*, and stored as a single document. This document was then re-run through *Atlas.ti* to identify the quotes relating to each subtheme. These were stored as text files, and the quotes which best represented the subthemes were selected as exemplary quotations. All themes, codes and categories were independently reviewed by DE.

## Results

### Sample

In total, 29 key AH informants provided data in the larger study. In this paper, we report on findings provided by 20 key informants who were rehabilitation professionals or managers of rehabilitation services (physiotherapists (*n* = 8), occupational therapists (*n* = 5), speech and language therapists (*n* = 2) and podiatrist (*n* = 1) and rehabilitation managers or directors (*n* = 4). Their different backgrounds and experiences in rehabilitation provided rich, heterogenous views of the need for improved capacity and how this could be achieved. The other nine key informants did not have a background in rehabilitation.

Themes, subthemes and verbatim supporting quotes are presented below.

#### Key theme 1: Content, purpose and construction of tools

##### An obvious gap

Participants’ responses consistently highlighted that lack of evidence-based tools (CPGs or other guidance documents) for rehabilitation at the primary care level in South Africa [4, 13, 20]. They also highlighted the unmet need for rehabilitation in South Africa, and the urgent need for tools to build capacity in writing and implementing rehabilitation CPGs specific to South African needs [1, 2, 4, 5, 13]. Participants noted that, despite the government’s intentions to re-engineer primary healthcare, there has been no or little input to improve provision of rehabilitation services at primary care level. Many participants were cautious that recommendations contained in CPGs would not be actionable within South African primary healthcare contexts because of a lack of capacity.

##### User-friendly and practical

There was common agreement that rehabilitation CPGs should be user friendly and practical to facilitate their uptake by over-burdened clinicians. Exemplar quotes come from a physiotherapist who was involved in CPG technical writing, and a rehabilitation manager. These quotes imply that extensive technical information in CPGs may not be useful, and that shorter, clinical friendly guidance (such as algorithms) may more efficiently enhance CPG uptake at primary healthcare level.

“*One of the big comments that we get about guidelines in this country which is consistent in fact every single guideline I’ve been involved in is that they’re too long and they’re not practical and they’re not usable. So one of the challenges of guidelines is awareness that they do exist for, for clinicians and making them simple and short enough.*” (ID 20, Physiotherapist – Technical writer)

“*Largely with making the treatment practical and relevant to our country in the context of their expertise and opinions essentially and the aim of a meeting such as that would be to produce a simple treatment algorithm which would be supported by the text.*” (ID 20, Physiotherapist – Technical writer)

“*The support that is needed in terms of associates to implement whatever guideline. If you are having a clinical guideline the assumption is that you have the resources to implement. If you need consumables the assumption is that consumables must be there but we don’t always have all the consumables needed.*” (ID 18, Rehabilitation director)

##### Integrated care and task transferability

A useable CPG at primary care level needs to reflect an integrated approach to care. None of the participants suggested the need for discipline-specific CPGs. Integrated care approaches are required to meet local healthcare systems constraints, as professional task ‘shifting’ is often needed due to the absence of multi-disciplinary teams at most primary care centres. For instance, a health professional from one AH discipline (say, physiotherapist) may be called upon to deliver care that would usually be provided by another AH professional (occupational therapist, speech and language therapist, or podiatrist) because someone from that discipline was not available at that specific facility.

##### Outcome measures

Participants highlighted the need to demonstrate the effectiveness of rehabilitation using appropriate outcome measures. Currently, there are no national or provincial databases that capture data on rehabilitation, largely because there have been more pressing health needs such as HIV or TB [2–4]. Participants recognised the need to collect standard rehabilitation outcomes to potentially illustrate the outcomes linked to CPG recommendations. If this occurred nationally, the information would be a powerful tool to advocate for improved access and equity in rehabilitation. Participants specifically suggested that outcome measures must be multi-disciplinary and holistic. This suggestion is in line with the person-centred approach current advocated by the South African Department of Health [22–24].

This occupational therapist’s quote captures the need for an integrated (multi-disciplinary) approach for South African rehabilitation CPGs and the importance of outcome measurement.

“*So, and I think again my understanding is that guidelines should be multi-disciplinary, so it should be for the whole team so that you’re looking at the outcomes as a whole and not just the OT* [occupational therapist] *outcomes or the physio or the medical or whatever, so it’s a whole team approach. Then maybe also giving links to other resources that the new graduates could consult for extra input if they needed it*.” (ID 15, Occupational therapist)

##### Local relevance

Participants in policy and management positions noted that, in South Africa, there was limited capacity to develop CPGs across all health areas, not just in rehabilitation. To counter this, guidance for priority programmes such as the HIV and TB had been adapted from CPGs developed by international institutions. Participants who are aware of this practice indicated the importance of contextualising CPGs to suit the national and provincial contexts. South Africa is a country with marked inequality and disease profiles that differ between geographical regions [4]. The following quote from a rehabilitation director indicates that, if CPGs are not adapted for local context, they may constrain CPG uptake at provincial and regional levels due to resource limitations and lack of relevance to local needs.

“*Some of the guidelines that we use are approved guidelines that come from the World Health Organization and then we adapt them through the national directorate and adapt them onto our province and use them and where we are having challenges are been reported back to national and provide feedback, we are not able to provide this with limited resources and when we need further training then we ask experts sometimes to be provided by the national department to come and assist in conducting training on how to implement guidelines that are coming from them.*” (ID 10, Rehabilitation director)

#### Key theme 2: Personal capacity

##### Developing individual capacity

This was mentioned many times as an essential step to help individuals to understand, write and implement CPGs. Personal empowerment was seen as essential to enhance individual and organisational uptake of South Africa-specific rehabilitation CPGs. Participants indicated that all rehabilitation stakeholders should be equipped with the knowledge, skills, willingness and confidence to at least use CPGs, if not to participate in writing them. However, from the interviews, it was clear that even basic understanding of what CPGs were was lacking. For instance, virtually all participants asked clarifying questions to determine if the interview was related to policy documents, protocols or guidelines. A quote by this rehabilitation manager highlights this issue:

“*Very few guidelines available at the moment. What exactly do you mean by a guideline?*” (ID 12, Physiotherapist – Rehabilitation manager)

##### Personal training

Personal training was viewed as an essential strategy to address individual knowledge and skill gaps. Some participants gave suggestions about training content such as providing basic information about guideline taxonomies. There was also a strong sense that training should also encompass implementation and subsequent monitoring. Continual professional development opportunities were suggested as a feasible pathway for training as this quote suggests.

“*I guess, I mean national associations are one, continuing professional development opportunities is the obvious one as facilitators of guideline implementation, …*” (ID 15, Occupational therapist)

##### Developing future trainers

Participants also noted that an important prerequisite to building capacity is the ongoing availability of experienced trainers. Therefore, the development of future trainers was suggested as an important aspect of building CPG capacity. Contingency planning is a particular challenge, considering the high staff turn-over rates within the public healthcare system. Building capacity not only in training but also of trainers to advocate for CPG use was viewed as a crucial element for sustainability.

“*In terms of going forward I am hoping that we will retain because people have developed quite a bit of skill in specifically in their specific areas around adaptation or contextualisation of guidelines, ...*” (ID 21, Physiotherapist, 2015)

“*So we brief them, we train them, we give them information and we all in agreement then they as a team in their own districts, they will cascade, all we do is support and monitor for those who do not have capacity to train or give out the information.*” (ID 8, AH – Dietician)

#### Key theme 3: Workforce capacity

##### Rehabilitation staff shortages

A common theme was the significant shortage of rehabilitation workers in South Africa. A rehabilitation manager responsible for planning rehabilitation services indicated that staff shortages are critical to address when building capacity in rehabilitation CPGs. To address insufficient workforce capacity, a multi-level workforce approach was suggested (as was also advocated by WHO [2, 3]). This could include rehabilitation professionals, vocationally trained community workers and mid-level rehabilitation workers, healthcare assistants, as well as clients and their carers. The quote below underscores the importance of mid-level workers and assistants, etc., who may alleviate current South African workforce shortages. This implies that, within South African contexts, it is essential for rehabilitation CPGs to consider all stakeholders who provide rehabilitation.

“*Over the last year or so we’ve tried to change that, so you will see now in the primary healthcare platform more and more allied health practitioners have been employed at the sub-district level, we are now piloting these mid-level workers for rehabilitation care workers and now have a technical working group that is looking at this thing.*” (ID22b, Rehabilitation director)

The consequences of workforce shortages manifest at two levels.

##### Time for self-change

Having dedicated time was noted by many as essential to ensure effective individual uptake of CPGs. It was also acknowledged that putting CPGs into place may reduce workplace pressures and improve patient flow, because care may be more effective and efficient. However, tensions were noted between finding time to locate and read CPGs, and to implement change, whilst dealing with the continual pressures of high patient volume and high patient to staff ratios.

##### Time to educate and influence others

Time was also required when influencing and transferring skills to colleagues at different levels of care. Making changes within departments, and within organisations, required time as well as trained personnel. The quotes below from rehabilitation managers illustrate these issues.

“*Barriers* [for guideline implementation] *would be, which is one I hate to admit, lack of staff. You know, you just don’t have the staff available.*” (ID 12, Rehabilitation manager)

“*Staff turnover, new people and the people that you train they usually don’t cascade the information as well because they won’t be having time.*” (ID 8, Manager)

##### Lack of basic infrastructure

Implications for CPG implementation are that efforts should be rewarded, and the interventions sustainable. This can refer to workforce, consumables, financial resources, effort and goodwill, and behaviour changes. As this rehabilitation director noted:

“*The support that is needed in terms of associates to implement whatever guideline. If you are having a clinical guideline the assumption is that you have the resources to implement. If you need consumables the assumption is that consumables must be there but we don’t always have all the consumables needed.*” (ID 18, Rehabilitation director)

#### Key theme 4: Systems capacity

##### Understanding rehabilitation

Participants indicated that a poor understanding of rehabilitation by other healthcare providers may be a factor influencing the uptake of CPGs at primary care level. This may well be related to overt historical focus on mortality from communicable diseases in South Africa [4] and the lack of system readiness to provide for people who are now living with chronic communicable and non-communicable diseases. The quote below from a rehabilitation manager outlines this issue.

“*The doctors, our managers, the rest of the team needs to buy in to this to understand what we’re doing. They need to obviously, I would love for them to contribute to what we’re doing.*” (ID 19B, Rehabilitation manager)

##### Rehabilitation across all levels of care

Seamless integration of rehabilitation into all levels of care (from primary to quaternary) must be addressed, as participants noted that rehabilitation services seem to be biased towards the higher levels of care. Primary healthcare is seen as an inexpensive, client-focused care level which, if better resourced, could deal with many more patients, more effectively. The current fragmented rehabilitation service delivery approach is not efficient or effective, as one rehabilitation director suggested.

“*Healthcare 2010 and Healthcare 2030 we’ve always identified rehabilitation and not just in the Western Cape, nationally as well but we have never ever put our money where our mouth is and as a result the rehabilitation services are fragmented, they are largely in the metro and in the tertiary hospitals.*” (ID 22, Rehabilitation director)

##### Communication

The importance of communication was highlighted, between different levels of care, provinces, team members, professional associations, and educational and government institutions. A physiotherapist involved in CPG development indicated that CPG implementation could be facilitated via good dialogue with professional societies (regarding coordination of activities, advocating for CPGs and financial support for research):

“*So the support that we had from the Society through the consultant really helped because I mean if I had to be the only person kind of driving it.*” (ID 21, Physiotherapist)

##### Professional support

In South Africa, all newly qualified therapists are required to do 1 year of community service. These community placements are usually within rural and remote areas, and the new graduates are often sole practitioners. CPGs would assist them in delivering evidence-based rehabilitation, particularly if they were supported by good communication with mentors and were involved in supportive collaborative partnerships. The speech and language therapist below suggests that CPGs can also play a role in supporting new graduates who are often working independently in remote regions, and with little guidance.

“*So firstly, it’s equipping them with knowledge and skill to deal with that. So that’s one. But actually, there’s very little support provided for people in com-serve* [community service] *for that kind of thing*.” (ID 23, Speech and language therapist)

##### Collaborations/partnerships

The need to work collaboratively with government departments, educational institutions, and healthcare managers and clinicians was regularly expressed. Leveraging off combined resources and diverse skill sets, the development and implementation of good quality CPGs can be facilitated in a way that met different professional agendas. However, concern was expressed about the lack of productive partnerships due to issues such as tensions between clinicians and academics.

“*We struggle to find people that can sort of lead the profession in development of guidelines and implementation of guidelines, because it’s like the clinicians on one side and the academics on the other side.*” (ID 13, Physiotherapist – South African Society of Physiotherapy)

##### Financial resources

Lack of financial resources for CPG writing, training and implementing was perceived to be a major barrier for CPG use in rehabilitation. Some of the participants suggested that guaranteed central funding streams were required so that CPG activity could be planned and have tangible, achievable timelines.

“*Look, there are a number of ways, one where what we are able to do, nationally, we can bring together people to be together in the way of training the trainer but for more localised training some province might be able to have their own provincial and fund it but what the, the likely to be able to fund would be a facilitation fee and then institutions, institutions pay for travel, accommodation but the province is able to pay for facilitation and the venue*.” (ID 18, Rehabilitation director)

##### Deductive analysis

Table 1 presents the four themes and 17 subthemes identified from the interviews, mapped to the Potter and Brough framework of nine elements within a hierarchy of capacity-building needs [7]. Each subtheme mapped to at least one Potter and Brough element, and it was clear that the interactivity proposed between hierarchy levels in the Potter and Brough model could also be anticipated between our findings. Without sound systems in place, for instance, workforce and personal capacity could not be developed, and these in turn led to contextualised CPG construction. An example of this is that the need for systems capacity was required in 16 of our 17 subthemes, highlighting the importance of sound systems to underpin growth of personal, workforce and contextual capacity. On the other hand, CPGs which were user friendly and practical, and which underpinned integrated care and task transferability, required higher order capacity development elements of skills and tools (which assumed that sound systems, structures, roles, staff and infrastructure were already in place).

**Table 1:**
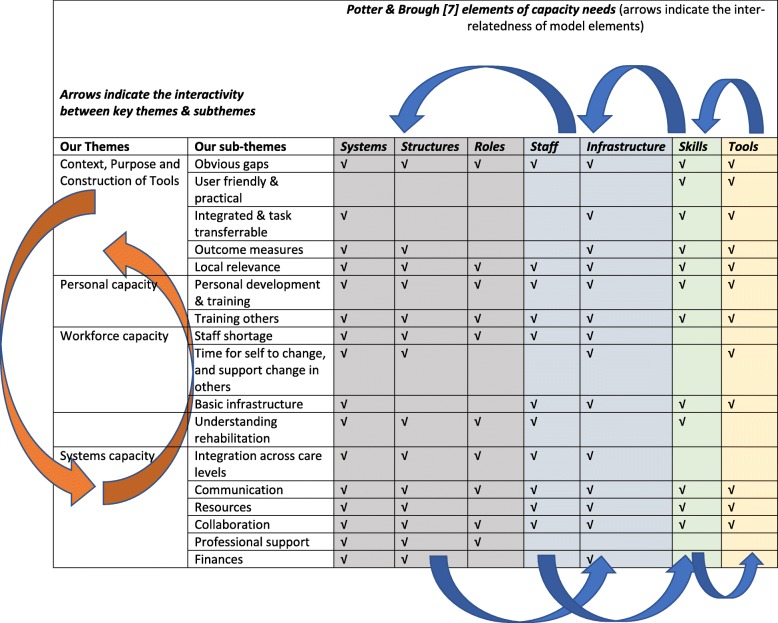
Our themes and subthemes mapped to the Potter & Brough framework [7]

Developed from this mapping process was a set of principles and strategies specific to South African rehabilitation capacity-building to better use contextually relevant rehabilitation CPGs. These principles and strategies could underpin discussions about the delivery of cost-effective, equitable and efficient rehabilitation, improve the lives of individuals with disability, and enable provincial and national governments to meet chronic healthcare targets. The principles and strategies are outlined in Table 2.

**Table 2:** Principles and strategies

Principle	Strategies
1. Construct, formalise and resource integrated national networks of rehabilitation stakeholders to ensure contextually relevant action on rehabilitation	• Formalise networks that equitably represent all rehabilitation stakeholders (policy-makers, funders, researchers, managers, clinicians, patients and families) • Seek committed, ongoing engagement of, and collaborations between, rehabilitation stakeholders • Identify change champions at all levels of government, and healthcare settings, who will promote person-centred rehabilitation that optimises quality of life and contribution to family, community, province and country • Identify and agree on standard methods of communication and reporting between stakeholders to ensure that important rehabilitation messages are heard by those who can action change
2. Determine and provide the resources required to produce and implement evidence-based, contextually relevant rehabilitation CPGs	• Recognise the costs required to write and implement clinical practice guidelines (CPGs) at government and organisational level • Develop a national plan of action that promotes efficient CPG writing and supports implementation and evaluation • Promote the notion that all rehabilitation CPGs should have implementation plans which should be auditable • Resource a central agency to produce high-quality rehabilitation CPGs for priority chronic communicable and non-communicable diseases • Develop standard reporting templates on CPG implementation • Develop standard methods of capturing rehabilitation outcomes
3. Provide ongoing training to rehabilitation stakeholders on CPG implementation and evaluation strategies	• Raise awareness of the concept of rehabilitation at all levels of government, as well as for medical and nursing disciplines, at organisational, department and clinic levels to ensure improved and shared understanding of the purpose and potential impact of rehabilitation • Provide training on how to implement rehabilitation CPGs at all levels of government, as well as at organisational, department and clinic levels to ensure that implementation plans of rehabilitation CPGs can be followed across the nation • Engage educational institutions to promote the methods of construction, and use of, rehabilitation CPGs to students
4. Invest in workforce redesign to ensure equity of access to evidence-based rehabilitation care	• Develop an agreed workplace hierarchy of competencies, roles and responsibilities for South African rehabilitation workers • Establish standard training requirements and competencies for all rehabilitation workers • Develop career pathways for rehabilitation workers • Identify and address rehabilitation workforce gaps • Identify ways in which patients and families can become part of the rehabilitation workforce

## Discussion

This is the first paper, as far as we are aware, that explores capacity development in rehabilitation CPGs from the perspectives of national stakeholders in a country with huge disability burden rehabilitation. The application of a deductive methodology building on an established capacity-building framework [7] enabled us to produce novel principles and strategies that could improve South African national, provincial and regional capacity in rehabilitation. High-quality, locally relevant CPGs are the vehicle that could support the delivery of high-quality rehabilitation for all South Africans who require care.

The strengths of the study were its nationally representative sample of key informants with views to share about capacity-building in rehabilitation in South Africa. We attempted to develop a sampling framework that allowed us to capture as many representative rehabilitation stakeholder voices as possible in the complex environment that is South African primary healthcare. However, we may not have captured all relevant perspectives. We also applied a structured deductive framework to assist us to make sense of the rich interview data, and to produce summary information that could guide capacity-building around South African rehabilitation CPGs. We chose an internationally tested framework that appeared to resonate with our context, aims and data [6]. Despite this, the choice of another framework might have facilitated different deductions, summaries, and capacity-building principles and strategies.

The design, practicality and local relevance of rehabilitation CPGs were highlighted as essential elements to improve CPG uptake in primary care. This concurs with published reports that state that barriers to CPG use are the length of guidelines, the lack of relevant strategies and facilitation for implementation and environment, and organisational constraints [25]. These findings suggest that a pragmatic approach to CPG capacity-building is needed. This could be spearheaded by a well-resourced central CPG writing agency where local context could be taken into consideration. As identified in the interviews, local factors relate to resources, available workforce, workforce training, facilities, collaborations and communication. In addition to these local considerations, our participants also indicated that CPG recommendations should be relevant for all stakeholders who will either use or drive the use of CPGs locally. These stakeholders should include community workers, family members and carers who play a vital role in rehabilitation due to current dire shortages in the rehabilitation workforce. Inclusion of all potential stakeholders is in line with the WHO recommendations for a multi-level workforce framework [2, 3].

Contextualisation of CPGs requires methodological knowledge and skills that are not as well understood as de novo CPG development skills [25–27]. However, as contextualisation is critical to CPG implementation within the South African context, a cohort of methodologists who are trained and experienced in these approaches is needed. In South Africa, and other countries with constrained resources, opportunities to upskill and train individuals in new and emerging CPG methodologies are limited. These emerging methods include adopting, contextualising, adapting and updating existing good quality CPGs [11, 28–30]. Opportunities for efficient training should be identified, particularly with the aim of promoting innovative CPG writing skills, producing contextualised recommendations, and establishing networks. One training example is a set of free online resources, the South Africa-contextualised CPG Development Toolkit [29], which is a comprehensive CPG resource designed for South African users.

At systems level in South Africa, a major implementation challenge will be to advocate for better integration of rehabilitation into primary care systems. The South African National Department of Health is redesigning its approach towards an integrated healthcare system (as opposed to the traditional specialist/expert clinics) [5, 24]. Our findings show that collaborations between different stakeholders (including health professional associations and non-government organisations) will be crucial to encourage the use of rehabilitation CPGs at the primary care level. Such collaborations could ensure continuity of care between health sectors and enhance communication between all levels and types of care. These collaborations will also be essential to facilitate inter-professional care delivered in a cost-effective systems approach. For example, whilst it has strong evidence of effectiveness internationally, employing multidisciplinary rehabilitation teams in primary healthcare facilities is not feasible in South Africa within the near future because of scarce workforce and funding. Therefore, contextualised CPG recommendations must include feasible implementation plans that can be executed within contextual barriers.

Capacity development linked to collaboration with higher education institutions is a potentially useful strategy to facilitate the uptake of CPGs. Such collaboration is also important for supporting local research in the fields of CPG contextualisation and new writing methods. Higher education institutions can become involved in CPG training as a potential avenue to upskill local rehabilitation professions and meet enrolment and income generation targets. These CPD activities should be affordable, particularly to therapists working at primary care level with the public sector. CPD activities should be well coordinated to prevent fragmentation and resource wastage, which will hamper progress in building CPG capacity. Training programmes should be comprehensive and include strategies to implement, monitor and evaluate the use of CPGs. Such national data will be essential to gain support from health policy-makers.

Understanding, accessing and implementing rehabilitation CPGs in South Africa will continue to be a challenge for all health professionals, unless the suggested principles and strategies can be actioned [4, 24, 25]. The participants in our study demonstrated how much guidance was required on what constituted a good CPG, how to disseminate and implement them, or evaluate their impact. For instance, resource constraints in South Africa mean that paper-copy dissemination is expensive, and copies may not reach their target. The challenge with electronic dissemination is a lack of information technology or unstable internet access in many rural or remote regions.

## Conclusion

Capacity-building in CPG implementation and uptake is crucial in rehabilitation, particularly in the South African context. South African rehabilitation services can benefit from a multi-level CPG capacity-building approach focusing on the performance, personal, workforce and systems levels. However, challenges in building capacity in CPG uptake at the South African primary care level are not unique to rehabilitation. Improved quality care using CPGs could be well addressed by a national steering body, tasked with initiating and managing a CPG repository in South Africa.
